# Occurrence of skin manifestations in patients of the Swiss Inflammatory Bowel Disease Cohort Study

**DOI:** 10.1371/journal.pone.0210436

**Published:** 2019-01-25

**Authors:** Nina Roth, Luc Biedermann, Nicolas Fournier, Matthias Butter, Stephan R. Vavricka, Alexander A. Navarini, Gerhard Rogler, Michael Scharl, on behalf of the Swiss IBD Cohort Study Group

**Affiliations:** 1 Department of Gastroenterology and Hepatology, University Hospital Zurich, University of Zurich, Zurich, Switzerland; 2 Department of Internal Medicine, Buergerspital Solothurn, Solothurn, Switzerland; 3 Institute of Social and Preventive Medicine (IUMSP), Lausanne University Hospital, Lausanne, Switzerland; 4 Department of Dermatology, University Hospital Zurich, Zurich, Switzerland; 5 Zurich Center for Integrative Human Physiology, University of Zurich, Zurich, Switzerland; Holbæk Hospital, DENMARK

## Abstract

**Background/Aims:**

Extraintestinal cutaneous manifestations of IBD represent a severe disease complication and an early and accurate treatment might positively influence the disease course. Using the patient collective of the Swiss IBD Cohort Study (SIBDCS), we analysed epidemiological as well as clinical factors being associated with the onset of pyoderma gangrenosum, erythema nodosum and aphthous ulcers in IBD patients.

**Methods:**

We included 3266 SIBDCs patients, 1840 with Crohn’s disease (CD) and 1426 with ulcerative colitis (UC) or IBD unclassified (IBDU) and analysed the association of cutaneous manifestations with age, age at diagnosis time, type of disease, gender, family history, HLA-allotype, smoking, intestinal disease activity, therapy and other extraintestinal manifestations (EIM).

**Results:**

354 CD patients and 136 UC/IBDU patients presented with skin manifestations at any time during their disease course. In both, CD and UC, female gender and younger age at IBD diagnosis were significantly associated with extraintestinal skin manifestations. For CD, we also detected a positive family history as associated factor. As an indicator of more intensive intestinal disease activity, patients with cutaneous manifestations of IBD needed more frequently therapy with antibiotics, steroids, immunomodulators and anti-TNF. Multivariate analysis revealed female gender, younger age at diagnosis and presence of other extraintestinal manifestations as factors being associated with skin EIM in IBD patients and anti-TNF as well as immunomodulatory treatment in CD patients.

**Conclusion:**

Our results suggest that young females with a positive family history of IBD might be at increased risk for the onset of skin manifestations and require a careful screening for such complications.

## Introduction

Inflammatory bowel disease (IBD) patients are commonly affected by extraintestinal manifestations (EIM) affecting the joints, skin, eyes, and biliary ducts, the overall appearance ranges from 6% to 47% [1–9] and up to 10% at the time of IBD diagnosis [10]. Peripheral arthritis, erythema nodosum and aphtous ulcers are associated with the activity of the intestinal disease. Other EIM, such as primary sclerosing cholangitis (PSC), pyoderma gangrenosum, uveitis and spondylarthropathy, are considered independent from intestinal activity [1, 11–13]. We have recently shown that in 25.8% of patients of the Swiss IBD Cohort Study (SIBDCS) EIM occur even before IBD is diagnosed [14].

Previous studies showed that almost one quarter of patients with EIM present with a combination of several EIMs suggesting that the appearance of one EIM favours the onset of at least one other EIM [15, 16]. In the SIBDCS this constellation occurred in 15% of Crohn’s disease (CD) and 8% of ulcerative colitis (UC) patients [11]. Most patients with EIM present with a severe colitis and some of them also reveal a positive family history for IBD [15, 17]. This allows the assumption, that there is at least some genetic influence.

The inflamed intestinal mucosa may trigger an extraintestinal immune response due to specific epitopes that are detectable in, e.g. intestinal bacteria and synovia. The immune system is incapable of distinguishing the two epitopes and therefore attacks both, as shown by Bhagat et al. in 1994. They found an isoform of tropomysin, which is expressed in all the organs that can be affected by EIM of IBD as well as in the gut and postulated an autoimmune reaction towards this molecule as a cause of EIM in IBD patients [18]. Genetic factors may also critically influence this particular immune reaction. EIM occur more often in people carrying specific HLA-constellations, for example CD with HLA-A2, HLA-DR1 and HLA-DQw5; UC with HLA-DR103 [19]. There are even specific HLA-types associated with certain EIM. HLA-DRB1*0103, HLA-B*27 and HLA-B*58 are associated with erythema nodosum, spondylarthropathy and uveitis [20, 21]. One gene has already been associated with the risk of skin manifestation in IBD patients, namely TRAF3IP2 [22]. Further hints that genetic factors may influence the occurrence of erythema nodosum are provided by the fact that it is more frequently found in CD and female patients [2, 11, 23, 24].

From a clinical point of view, it would be important to know the patient characteristics that are associated with the onset of each single cutaneous manifestation in IBD patients. Therefore, using the exclusive patient collective of the SIBDCS, we studied the correlation between epidemiological and clinical factors and the development of erythema nodosum, pyoderma gangrenosum or aphthous ulcers in IBD patients.

## Patients and methods

### Patient data

Data were retrieved from the nationwide Swiss Inflammatory Bowel Disease Cohort Study (SIBDCS). The SIBDCS is a multicenter prospective observational population-based study and includes patients with IBD from Switzerland. The study was implemented in all regions of Switzerland in 2006 in a multidisciplinary effort by gastroenterologists, pathologists, psychologists and bioinformatics specialists. The cohort study is funded by the Swiss National Science Foundation (SNSF). For inclusion in the SIBDCS, all patients must have a diagnosis established at least 4 months prior to inclusion. Data are prospectively collected once a year and entered into a central database. Inclusion and exclusion criteria are described elsewhere [25]. A total of 3266 patients were included in the current study of which 1840 suffered from CD and 1426 from UC or IBDU. The last two were treated as one group.

### Study design

We included all patients involved in the SIBDCS [suffering from either CD, UC or IBD unclassified (IBDU) = indeterminate colitis (IC)] available in the cohort for our study and discriminated the patients into two groups, one group with skin manifestations (erythema nodosum, pyoderma gangrenosum or aphthous ulcers) at inclusion or during any follow-up and another group without skin manifestations at any time of their disease course. Only those three skin EIM are mentioned in the SIBDC questionnaires and therefore considered for further analysis. No other skin EIM, such as atopic dermatitis, were included in our analysis, since they would not reflect a predefined answer in the SIBDCS questionnaires. The skin manifestations were diagnosed by the treating physician who was usually a gastroenterologist. In case of uncertainties, the patients had been sent to a dermatologist for final diagnosis. This means, mostly the diagnosis has been made by clinical judgement rather than by a biopsy.

Patients with CD were considered as a single entity and patients with Crohn’s colitis and Crohn’s ileitis were not distinguished. UC and IBDU were grouped together. Univariate and multivariate logistic regression analyses were performed. The following possible explanatory variables were considered: 1) Epidemiological characteristics: diagnosis of CD, UC or IBDU, age, disease duration (years), gender, age at diagnosis and latest follow-up, body mass index (BMI), smoking status and family history of IBD. 2) Disease characteristics and complications: activity index, initial and current disease location, extra-intestinal manifestations (EIM), existence of CD-related complications such as stenosis, fistula, fissure, abscess, intestinal surgery, anemia and vitamin B12 levels. 3) Selected medications: 5-ASA, antibiotics, steroids, immunomodulators (azathioprine, 6-mercaptopurin), anti-TNF antibodies and calcineurin inhibitors. 4) Genetic data for skin EIM in CD and UC patients. 5) Longitudinal data on skin EIM in CD and UC patients.

Non-skin EIM included arthritis, uveitis/ iritis, ankylosing spondylitis and PSC. Malabsorption syndrome with consequent anemia and vitamin B12 deficiency was also considered as a complication. To assess disease activity and allow comparison between UC (Modified Truelove and Witts activity index, MTWAI) and CD (Crohn’s disease activity index, CDAI), disease activity measures were normalized to a value between 0 and 100 and expressed as an activity index. In particular, IBD medication was documented by the treating physician in the specific SIBDC questionnaires at patient inclusion and follow-ups as well as by the patient questionnaires.

### Statistical analysis

All statistical analyses were carried out using the Stata Software (v. 14.2, StataCorp, College Station, TX, USA) and the R software (v. 3.3.1, The R Foundation for Statistical Computing, Vienna, Austria). Normal QQ-plots were used to assess distribution of continuous data. Gaussian-distributed data were reported as mean, standard deviation and range, while non-Gaussian data were presented as median, interquartile range and range. Differences in means between two independent groups for Gaussian-distributed data were assessed using the Student’s t-test. Differences in distribution locations between two independent groups for non-Gaussian data were assessed using the Mann-Whitney-Wilcoxon ranksum test. Categorical data were presented as raw frequencies and relative percentages. Differences in distributions for categorical data between two or more groups were assessed using the Chi-square test, or the Fisher’s exact test in case of insufficient sample size.

Time-to-event data were analysed using the Kaplan-Meier estimator, using specific techniques to deal with interval-censored data. Results were presented as cumulative proportion curves.

Multivariate logistic regression was used to assess the association of multiple factors with the occurrence of skin EIM. First, all factors with univariate p-value < 0.200 were included into the multivariate model. Non-significant factors (p>0.05) were then excluded from the model one-by-one, until all remaining factors were significant. In a last step, all factors that were left aside were once more inserted one-by-one into the model and kept if proven significant, while checking for model consistency at each step of the procedure.

### Ethical considerations

The IBD cohort study has been approved by the respective ethics committees in Switzerland (Ethics Committee of the Canton Zürich: EK-1316). All patients signed an informed consent and confirmed their participation in the cohort study at the time of enrolment and gave informed consent for data collection and analysis for research purposes. The current substudy has been evaluated and approved by the scientific board of SIBDCS.

## Results

### Epidemiology of skin manifestations in SIBDCS patients

In our study, we analysed data from 3266 IBD patients, of whom 1840 suffered from CD and 1426 from UC/IBDU. Out of the 1840 CD patients, 354 (19.2%) patients suffered from skin manifestations at any time during their disease course. Out of the 1426 UC/IBDU patients, 136 (9.5%) patients suffered from skin manifestations at any time during their disease course. For both, CD and UC/IBDU patients, the median age at occurrence of the skin EIM was 39 years. In 222 (62.7%) of the CD patients and in 94 (69.1%) of UC/IBDU patients, the skin EIM had already occurred before inclusion into the SIBDCS. With respect to CD patients, 29 (1.6%) suffered from pyoderma gangrenosum, 140 (7.6%) from erythema nodosum and 238 (12.9%) from aphthous ulcers. With respect to UC/IBDU patients, 25 (1.8%) exhibited pyoderma gangrenosum, 51 (3.6%) erythema nodosum and 77 (5.4%) aphthous ulcers (Table 1).

**Table 1:** Skin manifestations (pyoderma gangrenosum, erythema nodosum, aphthous ulcers) in SIBDCS patients.

	CD patients	UC/IBDU patients	
Skin EIM	n = 1840	n = 1426	
**No**	1486 (80.8)	1290 (90.5)	2776 (85.0)
**Yes**	354 (19.2)	136 (9.5)	490 (15.0)
**Pyoderma gang.**	29 (1.6)	25 81.8)	54 (1.7)
**Erythema nod.**	140 (7.6)	51 (3.6)	191 (5.9)
**Aphtous ulcers**	238 (12.9)	77 (5.4)	315 (9.6)
**Pyoderma gang. Occurence**			
**Before inclusion**	20 (69.0)	20 (80.0)	40 (74.1)
**After inclusion**	9 (31.0)	5 (20.0)	14 (25.9)
**Erythema nod. Occurence**			
**Before inclusion**	95 (67.9)	38 (74.5)	133 (69.9)
**After inclusion**	45 (32.1)	13 (25.5)	58 (30.4)
**Aphtous ulcers Occurence**			
**Before inclusion**	133 (55.9)	48 (62.3)	181 (57.5)
**After inclusion**	105 (44.1)	29 (37.7)	134 (42.5)
**Skin EIM Occurence**			
**Before inclusion**	222 (62.7)	64 (69.1)	316 (64.5)
**After inclusion**	132 (37.3)	42 (30.9)	174 (35.5)
**Age at Skin EIM Occurence (years)**			
**median, IQR, range**	39, 27–49, 11–73	39, 30–51, 18–79	39, 28–50,11–79

### Disease characteristics, complications and skin manifestations in CD patients

242 (68.4%) of CD patients were female, significantly more than male (112, 31.6%; (p = <0.001). In the group without skin manifestations no difference between genders (51.5% male vs. 48.5% female) was detected. Furthermore, those patients with skin manifestations were significantly younger at CD diagnosis than those without skin manifestations (25.8 years vs. 27 years, p = 0.006). However, no significant difference in age at latest follow up, disease duration, BMI or smoking status at diagnosis and latest follow-up was found between the two groups. CD patients with skin manifestations at enrolment showed a significantly higher rate of positive family history for IBD (16.9% vs. 12.5%; p = 0.006). The family history of IBD was unknown in 6.8% of patients with skin manifestations and 11.5% of patients without skin manifestations (Table 2).

**Table 2:** Skin manifestations (pyoderma gangrenosum, erythema nodosum, aphthous ulcers) in relations to significant patient characteristics in epidemiology.

	No Skin EIM	Skin EIM	All CD patients	pvalue
**Number of patients**	1486 (80.8)	354 (19.2)	1840 (100.0)	
**Gender**				
**Male**	765 (51.5)	112 (31.6)	877 (47.7)	<0.001
**Female**	721 (4805)	242 (68.4)	963 (52.3)	
**Age at diagnosis [y] (median,IQR)**	27, 20–37, 3–81	25.8, 19–35, 1–74	26, 20–37, 1–81	0.006
**Age at latest follow-up [y]** **(median, IQR)**	44, 33–57, 16–94	43, 32–54, 18–78	44, 33–56, 16–94	0.171
**Disease duration [y] (median, IQR)**	13, 7–21, 0–57	13, 8–22, 0–47	13, 7–21, 0–57	0.13
**BMI at latest follow-up [kg/m2] (median, IQR)**	23, 20–26, 14–47	22, 21–25, 15–31	23, 20–26, 14–47	0.408
**Smoking status at diagnosis**				
**Non-smoker**	719 (48.4)	156 (52.5)	905 (49.2)	
**Smoker**	695 (46.8)	159 (44.9)	854 (46.4)	
**Unknown**	72 (4.9)	9 (2.5)	81 (4.4)	0.097
**Smoking status at latest follow up**				
**Non-Smoker**	1007 (67.8)	237 (67.0)	1244 (67.6)	
**Smoker**	469 (31.6)	114 (32.2)	583 (31.7)	
**Unknown**	10 (0.7)	3 (0.8)	13 (0.7)	0.832
**Family history of IBD**				
**None**	1129 (76.0)	270(76.3)	1399 (76.0)	
**Yes**	186 (12.5)	60 (16.9)	246 (13.4)	
**Unknown**	171 (11.5)	24 (6.8)	195 (10.6)	0.006

There was no significant difference in the initial disease location between patients who developed skin manifestations and those who did not in the course of observation. During the follow-up period a tendency towards a shift of the disease location from mainly in the ileocolon (L3, 44.9% vs. 27.5% without skin EIM; 42.7% vs. 24.6% with skin EIM) towards the terminal ileum (L1, 24.7% vs. 28.5 without skin EIM; 20.9% vs. 26.6% with skin EIM), colon only (L2, 18.8% vs. 29.7% without skin EIM; 24.3% vs. 32.3% with skin EIM) and upper gastro intestinal tract (L4, 0.7% vs. 2% without skin EIM; 0.9% vs. 4.5% with skin EIM) was detected. Considering the CDAI at enrolment in the SCIBD, there was a significantly higher disease activity in the patients group with vs. without skin manifestations (CDAI 51 vs. 31, p = <0.001). This difference remained also significant at latest follow-up (CDAI 33 vs. 20, p = <0.001) (Table 3).

**Table 3:** Skin manifestations (pyoderma gangrenosum, erythema nodosum, aphthous ulcers) in relations to significant disease characteristics and complications in CD patients.

	No Skin EIM	Skin EIM	All CD patients	pvalue
**Number of patients**	1486 (80.8)	354 (19.2)	1840 (100.0)	
**Initial disease location**				
**L1**	367 (24.7)	74 (20.9)	441 (24.0)	
**L2**	279 (18.8)	86 (24.3)	365 (19.8)	
**L3**	667 (44.9)	151 (42.7)	818 (44.5)	
**L4 only**	11 (0.7)	3 (0.9)	14 (0.8)	
**Unclear/Unknown**	162 (10.9)	40 (11.3)	202 (11.0)	0.156
**Last disease location**				
**L1**	424 (28.5)	94 (26.6)	518 (28.2)	
**L2**	442 (29.7)	114 (32.2)	556 (30.2)	
**L3**	409 (27.5)	87 (24.6)	496 (27.0)	
**L4 only**	30 (2.0)	16 (4.5)	46 (2.5)	
**Unclear/Unknown**	181 (12.2)	43 (12.2)	224 (12.2)	0.06
**CDAI at enrolment (median, IQR)**	31, 11–49, 0–323	51, 20–96, 0–450	34, 12–76, 0–450	<0.001
**CDAI at latest follow-up (median, IQR)**	20, 6–47, 0–235	33, 8–65, 0–345	23, 6–50, 0–345	<0.001
**CD-related complications**				
**Perianal Fistula**	372 (25.0)	108 (29.7)	477 (25.9)	0.074
**Other Fistula**	233 (15.7)	61 (17.2)	294 (16.0)	0.474
**Any Fistula**	207 (34.1)	132 (37.3)	639 (34.7)	0.26
**Abscess**	351 (23.6)	102 (28.8)	453 (24.6)	0.042
**Stenosis**	638 (42.9)	1545 (43.8)	793 (43.1)	0.771
**CD-related surgery**				
**Intestinal resection**	608 (40.9)	152 (42.9)	760 (41.3)	0.487
**Fistula/Abscess surgery**	368 (24.8)	92 (26.0)	460 (25.0)	0.633
**Any Surgery**	758 (51.0)	184 (52.0)	942 (51.2)	0.743
**Anemia**				
**During SIBDCS follow-up**	421 (28.9)	125 (35.5)	546 (30.2)	0.016
**At latest follow-up**	142 (11.9)	30 (10.5)	172 (11.6)	0.506
**Vit. B12 level at latest follow-up [pmo]/l) (median, IQR)**	241, 176–323, 33–2435	252, 188–354, 28–1319	243, 178–328, 28–2435	0.164
**Ever received Vit. B12 Suppl.**	595 (49.8)	173 (56.0)	768 (51.1)	0.052
**Extraintestinal manifestations**				
**Arthritis**	622 (41.9)	264 (74.6)	886 (48.2)	<0.001
**Uveitis/Iritis**	117 (7.9)	88 (24.9)	205 (11.1)	<0.001
**Ankylosing spondylitis**	85 (5.7)	41 (11.6)	126 (6.8)	<0.001
**PSC**	10 (0.7)	2 (0.6)	12 (0.7)	1
**Any of the above**	673 (42.3)	274 (77.4)	947 (51.5)	<0.001

Considering CD-related complications there was no difference between patients with or without skin manifestation during observation period for perianal fistula, any other fistula, abscess or stenosis. However, patients with initial skin manifestations suffered from a significantly higher rate of abscesses (28.8% vs. 23.6%, p = 0.042). As for CD-related surgery no significant difference for intestinal resection, fistula or surprisingly even abscess surgery (26% vs. 24.8%) could be detected. Although there was no significant difference between vitamin B12 levels (Median 252 pmol/l vs. 241 pmol/l), patients with skin manifestations tended to receive vitamin B12 supplementation more often (56% vs. 49.8%, p = 0.052). As for anemia those patients with skin manifestation developed significantly more anemia during SIBDCS follow up (35.5% vs. 28.9%, p = 0.016). Nevertheless, at latest follow-up there was no longer any difference detectable (10.5% vs. 11.9%, p = 0.506) (Table 3).

Finally, we analysed the association of other EIM with the presence of skin manifestations. We found significant associations of early skin manifestations with arthritis (74.6% vs. 41.9%, p = <0.001), uveitis or iritis (24.9% vs. 7.9%, p = <0.001), ankylosing spondylitis (11.6% vs. 5.7%, p = <0.001) and EIM in general (77.4% vs. 42.3%, p = <0.001). These details are shown in Table 3.

### Relation between treatment and skin manifestations in CD patients

We analysed data for treatment history and the onset of skin manifestation. We found a significant difference between the two groups. Patients with skin EIM used significantly more often antibiotics (24.6% vs. 16.3%, p = <0.001), steroids (91% vs. 85%, p = 0.003), immunomodulators (88.4% vs. 79.7%, p = <0.001) and anti-TNF antibodies (77.4% vs. 59.2%, p = <0.001), than patients without skin EIM. Furthermore, no differences for treatment with 5- ASA (60.7% vs. 56.9%) and calcineurin inhibitors (2.8% vs. 1.7%) was detected (Tables 4 and 5).

**Table 4:** Skin manifestations (pyoderma gangrenosum, erythema nodosum, aphthous ulcers) in relations to significant selected medication in Crohn’s patient; 5-ASA: Salazopyrin, EIM: extraintestinal manifestations.

	No Skin EIM	Skin EIM	All CD patients	pvalue
**Number of patients**	1486 (80.8)	354 (19.2)	1840 (100.0)	
**Treatment history**				
**5-ASA**	845 (56.9)	215 (60.7)	1060 (57.6)	0.185
**Antibiotics**	242 (16.3)	87 (24.6)	329 (17.9)	<0.001
**Steroids**	1263 (85.0)	322 (91.0)	1585 (86.1)	0.003
**Immunomodulators**	1185 (79.7)	313 (88.4)	1498 (81.4)	<0.001
**Anti-TNF**	879 (59.2)	274 (77.4)	1153 (62.7)	<0.001
**Cacineurin inhibitors**	26 (1.7)	10 (2.8)	36 (2.0)	0.189

**Table 5:** Multivariate logistic regression results for CD patients.

Outcome: Skin EIM	OR (95%CI)	pvalue
**Gender**		
Male (ref)	1 (ref)	
Female	1.701 (1.173–2.468)	0.005
**Age at diagnosis** [per year]	0.981 (0.966–0.995)	0.009
**CDAI at latest follow-up** [per CDAI point]	1.005 (1.001–1.008)	0.006
**Immunomodulator treatment**		
Never (ref)	1 (ref)	
Yes	2.123 (1.035–4.358)	0.04
**Anti-TNF treatment**		
Never (ref)	1 (ref)	
Yes	1.762 (1.146–2.708)	0.01
**Other EIM**		
No (ref)	1 (ref)	
Yes	4.452 (2.813–7.046)	<0.001

### Disease characteristics, complications and skin manifestations in UC/IBDU patients

Patients with UC and skin EIM were more often female (58.1% vs. 45%, p = 0.004) and also younger at the time of UC/IBDU diagnosis than UC patients without skin EIM (27 years vs. 31 years, p = 0.008). At the latest follow-up both groups were of comparable age (45 years vs. 46 years). Therefore, the observed disease duration in patients with skin manifestations was significantly longer than in the other group without skin manifestations (13 years vs. 11 years, p = 0.029). We found no difference between the two groups for BMI, smoking status or family history of IBD. However, a considerable fraction of patients tended to stop smoking during follow-up (300 smokers at diagnosis vs. 198 smokers at latest follow-up). Data are presented in Table 6.

**Table 6:** Skin manifestations (pyoderma gangrenosum, erythema nodosum, aphthous ulcers) in relation to significant patient characteristics in epidemiology.

	No Skin EIM	Skin EIM	All UC/IBDU pat.	pvalue
**Number of patients**	1290 (90.5)	136 (9.5)	1426 (100.0)	
**Gender**				
**Male**	709 (55.0)	57 (41.9)	766 (53.7)	
**Female**	581 (45.0)	79 (58.1)	660 (46.3)	0.004
**Age at diagnosis [y]** **(median, IQR)**	31, 23–41, 3–83	27, 21–38, 14–79	31, 23–41, 3–83	0.008
**Age at latest follow-up [y]** **(median, IQR)**	46, 36–57, 17–89	45, 35–56, 18–86	46, 36–57, 17–89	0.409
**Disease duration [y]** **(median, IQR)**	11, 6–18, 0–59	13, 8–22, 0–41	11, 6–18, 0–59	0.029
**BMI at latest follow-up [kg/m2]** **(median, IQR)**	24, 21–26, 18–37	27, 21–28, 21–31	24, 21–27, 18–37	0.49
**Smoking status at diagnosis**				
**Non-smoker**	966 (74.9)	106 (77.9)	1072 (75.2)	
**Smoker**	272 (21.1)	28 (20.6)	300 (21.0)	
**Unknown**	52 (4.0)	2 (1.2)	54 (3.8)	0.349
**Smoking status at latest follow up**				
**Non-Smoker**	1091 (84.6)	117 (86.0)	1208 (84.7)	
**Smoker**	180 (14.0)	18 (13.2)	198 (13.9)	
**Unknown**	19 (1.5)	1 (0.7)	20 (1.4)	0.912
**Family history of IBD**				
**None**	1020 (79.1)	106 (77.9)	1126 (79.0)	
**Yes**	131 (10.2)	20 (14.7)	151 (10.6)	
**Unknown**	139 (10.8)	10 (7.4)	149 (10.4.)	0.149

Initially most patients, independent from skin manifestations, had either a pancolitis (39.7% vs. 37.3%) or left- sided colitis (31.6% vs. 32.3%). The extent of the disease remained stable during the disease course. However, patients with skin EIM suffered from higher disease activity according to the MTWAI at enrolment (MTWAI 3 vs. 2, p = <0.001). IBD-related surgery as a result of several other complications was significantly more frequent in patients with skin EIM (18.4% vs. 10.2%, p = 0.003). Anemia was significantly more often observed in patients with skin EIM during any SIBDCS follow-up visit (42.6% vs. 29.7%, p = 0.002), but not at latest follow- up (15.8% vs. 13.8%, p = 0.573). Vitamin B12 levels did not differ between the two groups (285 pmol/l vs. 290 pmol/l), but those patients with skin EIM received significantly more often vitamin B12 supplementation (31.5% vs. 21.7%, p = 0.015).

Finally, skin manifestations were significantly more often associated with other EIM (75.7% vs 35.8%, p = <0.001) such as arthritis as the most frequent (68.4% vs. 30.3%, p = <0.001), uveitis/ iritis (16.2% vs. 4.5%, p = <0.001) and ankylosing spondylitis (7.4% vs. 2.5%, p = 0.001). No such association could be detected for PSC (5.9% vs. 4.2%, p = 0.356). Data are presented in Table 7.

**Table 7:** Skin manifestations (pyoderma gangrenosum, erythema nodosum, aphthous ulcers) in relations to significant disease characteristics and complications in patients with ulcerative colitis (UC)/ IBD unclassified (IBDU).

	No Skin EIM	Skin EIM	All UC/IBDU pat.	pvalue
**Number of patients**	1290 (90.5)	136 (9.5)	1426 (100.0)	
**Initial disease location**				
**Pancolitis**	481 (37.3)	54 (39.7)	535 (37.5)	
**Left-sided colitis**	416 (32.3)	43 (31.6)	459 (32.2)	
**Proctitis**	262 (20.3)	18 (13.2)	280(19.6)	
**Unclear/Unknown**	131 (10.2)	21 (15.4)	152 (10.7)	0.087
**Last disease location**				
**Pancolitis**	450 (34.9)	44 (32.4)	494 (34.6)	
**Left-sided colitis**	462 (35.8)	53 (39.0)	515 (36.1)	
**Proctitis**	230 (17.8)	21 (15.4)	251 (17.6)	
**Unclear/Unknown**	148 (11.5)	18 (13.2)	166 (11.6)	0.731
**MTWAI at enrolment** **(median, IQR)**	2, 1–5, 0–19	3, 2–7, 0–18	2, 1–5, 0–19	<0.001
**MTWAI at latest follow-up** **(median, IQR)**	2, 0–4, 0–18	2, 1–4, 0–18	2, 0–4, 0–18	0.015
**IBD-related surgery**	131 (10.2)	25 (18.4)	156 (10.9)	0.003
**Anemia**				
**During SIBDCS follow-up**	354 (29.7)	58 (42.6)	412 (31.0)	0.002
**at latest follow-up**	122 (13.8)	16 (15.8)	138 (14.0)	0.573
**Vit. B12 level at latest follow-up [pmol/l] (median,IQR)**	290, 215–379, 24–1696	285, 213–351, 41–1476	290, 215–374, 24–1696	0.584
**Ever received Vit. B12 Suppl.**	210 (21.7)	39 (31.5)	249 (22.8)	0.015
**Extraintestinal manifestations**				
**Arthritis**	391 (30.3)	93 (68.4)	484 (33.9)	<0.001
**Uveitis/Iritis**	58 (4.5)	22 (16.2)	80 (5.6)	<0.001
**Ankylosing Spondylitis**	32 (2.5)	10 (7.4)	42 (2.9)	0.001
**PSC**	54 (4.2)	8 (5.9)	62 (4.3)	0.356
**Any of the above**	462 (35.8)	103 (75.7)	565 (39.6)	<0.001

### Relation between treatment and skin manifestations in UC/IBDU patients

Looking at the treatment history of the compared groups, we discovered significantly more frequently a therapy with antibiotics (17.6% vs. 9.6%, p = 0.003), steroids (92.6% vs. 77.8%, p = <0.001), immunomodulators (75% vs. 59.5%, p = <0.001), anti-TNF antibodies (43.4% vs. 33.2%, p = 0.017) and calcineurin inhibitors (17.6% vs. 8.7%, p = 0.001) at enrolment in patients with skin EIM. No difference was found for the therapy with 5- ASA (95% vs. 95%, p = 0.956). Data are shown in Tables 8 and 9.

**Table 8:** Skin manifestations (pyoderma gangrenosum, erythema nosodusm, aphthous ulcers) in relations to significant selected medication in patient with UC/IBDU.

	No Skin EIM	Skin EIM	All UC/IBDU pat.	pvalue
**Number of patients**	1290 (90.5)	136 (9.5)	1426 (100.0)	
**Treatment history**				
5-ASA	1225 (95.0)	129 (95.0)	1354 (95.0)	0.956
Antibiotics	124 (9.6)	24 (17.6)	148 (10.4)	0.003
Steroids	1004 (77.8)	126 (92.6)	1130 (79.2)	<0.001
Immunomodulators	768 (59.5)	102 (75.0)	870 (61.0)	<0.001
Anti-TNF	428 (33.2)	59 (43.4)	487 (34.2)	0.017
Calcineurin inhibitors	112 (8.7)	24 (17.6)	136 (9.5)	0.001

**Table 9:** Multivariate logistic regression results for UC/IBDU patients.

Outcome: Skin EIM	OR (95%CI)	pvalue
**Gender**		
**Male (ref)**	1 (ref)	
**Female**	1587 (1.018–2.474)	0.041
**Age at diagnosis [per year]**	0.974 (0.956–0.993)	0.006
**Other EIM**		
**No (ref)**	1 (ref)	
**Yes**	4.048 (2.511–6.526)	<0.001

### Longitudinal data considering the onset of cutaneous EIM in CD and UC/IBDU patients

**Fig 1:**
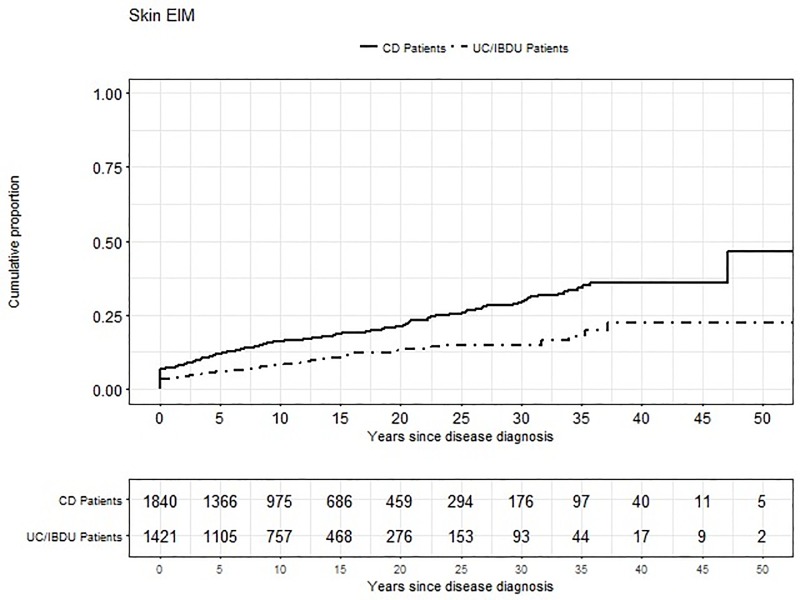
Cumulative proportion of skin manifestations over the years in Crohn’s patient (CD) and in patient with Ulcerative Colitis (UC)/ IBD unclassified (IBDU).

Finally, we assessed the longitudinal appearance of skin manifestation compared to both, other EIMs and first diagnosis of IBD (Fig 1). We found, that skin manifestations in either CD and UC/IBDU patients were the most frequent EIM of the disease. They were mainly reported as first EIM (75.5% vs. 87%) rather than as subsequent EIM (24.5% vs. 13.0%). After all, 23.6% of the skin manifestations in all IBD patients occur even before the diagnosis of the intestinal disease. This occurs slightly more often in CD patients than in UC patients (28.6% vs. 13%).

## Discussion

The aim of our study was to identify epidemiological and clinical factors being associated with the development of skin manifestations in patients with IBD. Our results revealed a higher prevalence of skin manifestations overall in female and young patients independent from the IBD subtype. This gender association has not been demonstrated for cutaneous EIM in general so far, although it was known from former studies, that especially erythema nodosum is associated predominantly with female gender [2, 11, 24, 26]. Further, a female predominance was generally known for familial IBD [27]. This might be due to some x-linked genes that are associated with autoimmune or chronic inflammatory diseases. For IBD there was so far only one such gene known called ARHGEF6 [22, 28]. For patients with CD, a family history of IBD was also highly associated with skin manifestations, while no such associations were detected for patients with UC. These results were inconsistent with those from former studies where no significant association of skin manifestations and family history could be observed for either UC or CD [26].

Our study demonstrated that IBD patients with skin EIM are in general younger at time of IBD diagnosis than IBD patients without skin EIM during their disease course. The onset of skin manifestations as well as a younger age at disease onset were both factors pointing towards a more severe disease course [29]. However, it must be pointed out that a younger age at diagnosis of patients with skin EIM (those patients are about 26 to 27 years at diagnosis) is not related to early onset IBD in pediatric patients. Smoking had also been associated with a severe disease course in CD patients, but with a milder course of disease in UC patients [30]. In CD, several studies showed more frequent relapses with higher use of steroids, immunomodulators and biologicals, as well as a higher rate of hospitalizations in smokers [31–34]. Compared to non-smokers, the risk of developing CD is increased fivefold in women and 1.3 fold in men [35]. However, smoking did not affect the onset of skin manifestations in CD or UC patients.

Erythema nodosum as well as aphthous ulcers had been correlated with intestinal disease activity in previous studies [36, 37]. Those observations point into the same direction as our data, since the patients with any of the three skin EIM (erythema nodosum, pyoderma gangrenosum and aphthous ulcers) revealed higher CDAI in CD and MTWAI in UC patients with skin manifestations in the SIBDC. These results were consistent with earlier studies showing that 70% of the patients with EIM presented with extensive intestinal disease, while patients without EIM present with a high intestinal disease activity in only 28% of the cases [10, 15, 38]. Severe intestinal disease can even be a predisposing factor for the onset of pyoderma gangrenosum [9, 38], regarding CD it favors the onset of erythema nodosum [39] and in the case of aphthous ulcers the connection to intestine is obviously given by anatomy. Therefore, the therapy of intestinal disease remains the primary therapy of the majority of cutaneous manifestations. Consequently, it is of high importance to treat the intestinal disease as early as possible and with high efficiency [13, 29, 40]. As in former studies, we found, that skin manifestations were in almost a quarter of the cases even reported before the diagnosis of the intestinal disease [14]. Their appearance should therefore be taken into account for daily clinical practice and patient care.

A limitation of the SIBDCS, however, is the fact that it is not fully population based as two third of SIBDCS patients are treated at tertiary center hospitals. Consequently, the study population consists of a large number of patients with a severe disease course, who are more likely to develop EIM. Nevertheless, the UC and CD patients with skin EIM in our study needed more antibiotic therapy, steroids, immunomodulators, anti-TNF antibodies and calcineurin inhibitors (UC patients only) than patients without skin EIM. This supported the observation that patients with EIM suffered often from a more severe disease course than patients without EIM (presence of EIM is a marker for a severe disease course itself). A further limitation of our study is that, due to the design of our study, we cannot identify a single skin manifestation that is driving the observed associations. Due our study design, we are only able to present associations between the onset of skin manifestations at any time during disease course and certain epidemiological or medical conditions. However, we are not able to provide risk factor analysis for the onset of skin EIM in our cohort. Such a risk factor analysis would be required to provide information about a possible, specific driver skin EIM in the patients. Also due to the design of our study and the SIBDCS, we are not able to provide sufficient information on treatment response of the skin manifestations or whether IBD treatment was changed based on the presentation of these skin manifestations.

Although according to our data it was difficult to verify, whether the increased use of biologics was due to the intestinal disease activity or the skin manifestation itself. For patients with UC and treated with the basic therapy 5–ASA, no difference between presence and absence of skin manifestations was found. This observation further indicated that skin manifestations occurred in patients with more severe disease. Interestingly and in good accordance to the above-mentioned findings, we detected a higher frequency of abscesses in CD patients in the group with skin manifestations. Although it must be mentioned that this higher frequency of abscesses did not result in a more frequent abscess surgery, the onset of abscesses alone can be already regarded as a marker for a severe CD disease course. We observed a higher rate of anemia in patients with skin manifestations in either CD or UC. These patients were substituted with vitamin B12 more often, although they had no vitamin B12 deficiency. In the course, anemia was no longer more frequent in patients with skin manifestations. We suggest that this was due to improved resorption of substrates after the resolution of intestinal inflammation through successful therapy.

We identified female gender and early onset of disease in UC and CD as well as a positive family history for skin manifestations in CD as epidemiological factors being associated with the development of skin manifestations in IBD patients. In the case of already known IBD, especially young female patients with high disease activity had a high probability also to develop skin manifestations. Our findings correlate with those from Ampuero et al in 2014 where female gender, young age at diagnosis, Crohn’s disease and the presence of other EIM were also identified as risk factors for skin manifestations in IBD. On the other hand they had shown that if biologicals were used early in the course of disease it prevented the development of skin manifestations [26]. For patients with such a constellation, special care should therefore be taken to detect the appearance of skin lesions as early as possible and to optimize patient care.

In summary, our data demonstrate that erythema nodosum, pyoderma gangrenosum, and aphthous ulcers in IBD patients are associated with a more severe disease phenotype. Further research will be needed to clarify the potential pathogenetic impact on the development of skin EIMs. This knowledge would be most helpful in the diagnosis and treatment of IBD patients and might help to stratify the patients according to their risk for developing skin manifestations and to initiate appropriate treatment.

## Abbreviations

CD: Crohn’s disease
CDAI: Crohn’s disease activity index
EIM: Extraintestinal manifestation
IBD: Inflammatory bowel disease
IBDU: Inflammatory bowel disease unclassified
IC: indeterminate colitis
MTWAI: Modified Truelove & Witts activity index
PSC: Primary sclerosing cholangitis
SIBDCS: Swiss Inflammatory Bowel Disease Cohort Study
SNP: Single nucleotide polymorphism
UC: Ulcerative colitis
